# Extraction of Microfibrillar Cellulose From Waste Paper by NaOH/Urethane Aqueous System and Its Utility in Removal of Lead from Contaminated Water

**DOI:** 10.3390/ma13122850

**Published:** 2020-06-25

**Authors:** Vadahanambi Sridhar, Hyun Park

**Affiliations:** 1 Global Core Research Centre for Ships and Offshore Plants (GCRC-SOP), Pusan National University, Busan 46241, Korea; sridhar@pusan.ac.kr; 2 Department of Naval Architecture and Ocean Engineering, Pusan National University, Busan 46241, Korea

**Keywords:** microfibrillated cellulose, waste paper, NaOH/urethane aqueous system, recycling, lead removal

## Abstract

Though recycling of waste paper is widely practiced but usually it is downgraded to lower valued recycled waste paper. Based on this concern, we report the development of novel NaOH/urethane aqueous system for extraction of microfibrillated cellulose from waste paper. The purity of so obtained microfibrillated cellulose (MFC) was evaluated by morphological tests using scanning electron microscopy (SEM) and transmission electron microscopy (TEM) and by evaluation of physicochemical properties using Fourier-transform infrared (FTIR) spectroscopy, X-ray diffraction (XRD), differential scanning calorimetry (DSC), and thermogravimetric analysis (TGA). Morphologies of MFC studied by SEM and TEM showed that the size of purified cellulose fibrils reduced when compared to that of waste paper but fibrils are cleaner and smoother due to the removal of talc and lignin. XRD analysis revealed that MFC exhibits good crystallinity. The utility of sulfonated and pristine microfibrillar cellulose in removal of lead from contaminated water is also reported. Our results show that renewable, sustainable, cheap, and waste biomass like waste paper can be used for producing valuable second-generation high-value products.

## 1. Introduction

With the population of earth crossing 7 billion, there is an ever-increasing demand for suitable materials to satisfy the world’s growing hunger for goods for which a wide range of metals, polymers, composites, etc. have been developed. Amongst these, synthetic and natural polymers play critical and ubiquitous roles in our everyday life. However, due to the growing concerns about end-of-life disposal of petroleum oil-based synthetic polymers, alternate biodegradable and biosourced plastics are rapidly gaining considerable interest. Amongst these, cellulose, the most abundant naturally occurring amphiphilic polymer in the biosphere, with an estimated annual production of 7.5 × 10^10^ to 1.5 × 10^12^ t [1,2], can be considered as an almost inexhaustible raw material source to satisfy the ever-increasing demand for environmentally friendly and biocompatible products. The unique physical, chemical, and electrical properties of cellulose make it as an excellent raw material in a wide range of products including paper, packaging, textiles, fibers, explosives, and, more recently, as stimuli-responsive actuators [3], sensors [4], etc. However, the applicability of cellulose in the abovementioned applications is highly dependent on purity, and plant-based cellulose, in general, is always associated with impurities such as hemicellulose, lignin, pectin, etc. Depending upon the source, the percentage of cellulose varies from as high as 90% in cotton to 40–50% in wood [5].

Despite the fact that cellulose is widely available, its insolubility in industrial solvents due to the extensive intrinsic hydrogen bonding, which forms a strong three-dimensional semicrystalline β-(1→4)-linked glucosyl units linear homopolysaccharide networks, makes its extraction difficult [6]. Traditional cellulose extraction technique like the industrially relevant “viscose process” is not only energy intensive but also has adverse environmental effect by releasing harmful chemicals like carbon sulfide, which is known to cause cancer, heart disease, birth defects, etc. In order to overcome these drawbacks, a wide range of techniques for cellulose extraction has been developed such as LiCl/dimethylacetamide [7], LiOH/urea [8], NaOH/urea [9], NaOH/thiourea [10], NaOH/urea/thiourea [11], N-Methylmorpholine N-oxide [12], ionic liquids [13], etc. Additionally, the morphology of cellulose extracted by abovementioned techniques is not uniform, and the end products are in micro- and nanoscales. Based on the morphology and aspect ratio of cellulose obtained a wide array of terms such as cellulose nanocrystals (CNC) [14], nanofibrillated cellulose (NFC) [15], bacterial nanocellulose (BNC) [16], cellulose nanoparticles (CNP) [17], microcrystalline cellulose (MCC) [18], cellulose microcrystallites (CMC) [19], microfibrillar cellulose (MFC) [20], etc. are in vogue. Of these, cellulose in micrometer scale are worth special mention since they are long, flexible, and can be processed with existing textile equipment. Though the abovementioned extraction systems are fairly effective in extraction of cellulose from high cellulose-containing raw materials such as expensive high-grade cotton linters, the efficacy of these chemicals in low-grade lignocellulosic biomass is less than satisfactory [7,12]. Amongst the lignocellulose raw materials, waste paper is worth a mention since literally, millions of tons of waste paper is produced annually, and though waste paper is widely recycled to low-grade recycled newspaper, i.e., cardboards for packaging, low-cost insulation tiles, etc., the value of recycled waste paper is considerably less than virgin paper. There are reports on extraction of cellulose from waste paper. Rodrigues Filho et. al. [21] converted waste paper to cellulose acetate, whereas Nguyen et. al. [22] used imidazolium-based ionic liquids to convert waste paper to cellulose aerogel. However, in all these aforementioned reports, cellulose was either converted to cellulose derivatives or the extraction of cellulose was carried out using expensive ionic liquids. Therefore, there is a need for development of economical cellulose extraction systems.

The purpose of this investigation is to report our discovery of NaOH/urethane aqueous system to extract microfibrillated cellulose from waste paper. It is widely reported that urea with two –NH_2_ groups is very effective in dissolution of cellulose in alkaline aqueous systems, we postulate that urethane, an ester of carbamic acid with a lone –NH_2_ moiety but considerably more reactive, can also efficiently dissolve cellulose in NaOH aqueous systems.

## 2. Experimental

### 2.1. Materials and Methods

Waste paper was collected from our office waste basket, whereas reagent-grade urethane (CAS number: 51-79-6, 99% purity), lead nitrate (CAS number: 10099-74-8, 99% purity, ACS reagent grade), p-toluenesulfonyl chloride (CAS number: 98-59-9, 99% purity, ReagentPlus grade), dimethyl acetamide (CAS number: 127-19-5, 99.8% purity, Anhydrous), lithium chloride (CAS number: 7447-41-8, 99% purity, ACS reagent), and sodium hydroxide (CAS number: 1310-73-2, 99.99% purity, semiconductor grade) were purchased from Sigma-Aldrich, Seoul, Korea, and were used as received. Waste paper was first shredded and boiled in deionized water for 30 min at 70 °C, washed with water to remove impurities, and then, dried overnight in a vacuum oven. NaOH and urethane were separately dissolved in deionized water to obtain 10 wt% urethane and 10 wt% NaOH aqueous solutions and subsequently mixed in deionized water to obtain 10 wt% NaOH/10 wt% urethane aqueous solutions. The dried waste paper pulp was added to the above NaOH/urethane solution, and the mixture was stirred for 180 min at room temperature after which, the reaction was quenched by adding cold deionized water to the reaction mixture and filtered by a vacuum filter. The solution was centrifuged at 5000 RPM for 30 min and subsequently dialyzed with deionized water until a constant pH was reached. The morphology was tested with a field-emitting scanning electron microscope (SEM) (Zeiss FEG-SEM Supra 25, Seoul, Korea) and transmission electron microscopic (TEM) images were recorded on TALOS F200X (Thermo Fisher Scientific Korea Ltd., Seoul, Korea), at operating voltage was 10 and 200 kV, respectively, in SEM and TEM. Fourier transform infrared spectra, FTIR, were recorded by a Thermo Scientific Nicolet iS10 Spectrometer (Thermo Fisher Scientific Korea Ltd., Seoul, Korea) with and attenuated total reflection (ATR) accessory, in the wave band range from 4000 to 400 cm^−1^. The spectra were acquired with a resolution of 4 cm^−1^ and a total of 32 scans. 1H-NMR spectra were recorded on a Bruker DRX-400 spectrometer (Bruker Korea Co., Ltd., Seongnam, Korea) at 400 MHz in CDCl_3_ solution. The chemical shifts were reported in parts per million (ppm) and with tetramethylsilane (TMS) as an internal standard. X-ray diffraction (XRD) patterns were recorded on Rigaku D-max diffractometer (Rigaku, Tokyo, Japan).

Batch type equilibrium adsorption experiments were performed using 0.1 mM Pb(II) solution prepared by dissolving Pb(NO_3_)_2_ in the background electrolyte at a controlled pH of 5.5 maintained by adding 0.01 M NaNO_3_. The choice of slightly acidic condition was chosen based on the fact that real-life experimental soils are in this pH range. In a typical experiment, a microfibrillated cellulose films (1 g L^−1^) were added to the solution and then, vigorously stirred at 400 rpm using a magnetic stirrer. The amount of lead adsorbed was measured by EDTA titration method as reported by Hisada et al. [23].

### 2.2. Qualitative Analysis

Chemical composition (α-celluloses, hemicellulose, and holocellulose) was estimated according to TAPPI 249-75 procedures. Briefly, 5 g of extracted microfibrillar cellulose was mixed with 500 mL of 7.5 wt% NaOH solution and stirred at 25 °C for 60 min. Precisely, 10 mL of 0.5 N potassium dichromate aqueous solution and 50 mL of concentrated sulfuric acid was added to 25 mL of the filtrate and stirred for 15 min. Finally, 50 mL of water and 2 drops of ferroin indicator were added and titrated against 0.1 N ferrous ammonium sulfate solutions until a purple color was obtained. For determination of holocellulose, 2 g of the sample was placed for 120 min in an oven maintained at 150 °C The percentage of α-celluloses, holocellulose, and lignin content were determined by the following equations:

(1) $$\alpha -cellulose\;\left(\%\right)=\frac{\left(6.85\times \left(V2-V1\right)\;\times N\times 20\right)\times 100}{A\times W}$$

and

(2) $$holo\;cellulose\;\left(\%\right)=\frac{A-B}{C}\times 100$$

where V1 and V2 are the volumes of filtrate and blank, N is the normality of ferrous ammonium sulfate solution, A is the volume of pup filtrate, W is dry weight of the sample, and A, B, and C are the oven dried weight of fibers, ash, and initial weights, respectively.

## 3. Results and Discussion

**Figure 1:**
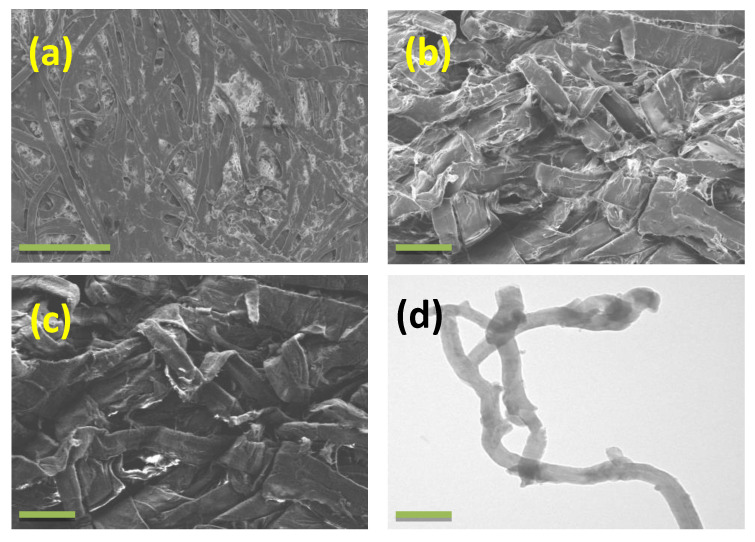
Scanning electron microscopy (SEM) micrographs of waste paper (**a**) and microfibrillated cellulose (**b**) extracted using NaOH/urethane aqueous system and corresponding secondary electron image (**c**), and representative transmission electron microscopy (TEM) image (**d**). Scale bars are 20 µm in (**a**), 2 µm in (**b**,**c**), and 1 µm in (**d**).

Figure 1a,b depicts the SEM morphology of waste paper and cellulose extracted by NaOH/urethane aqueous system, respectively. As can be observed in Figure 1a, the surface morphology of waste paper shows considerable amounts of micrometer-sized particulate impurities besides long fibrous cellulose-rich fibers on the surface.

These platy and layered structure impurities are probably talc and other clay materials that are added in traditional paper making to reduce tackiness and increase hydrophobicity. After treatment with our NaOH/urethane aqueous system, SEM micrographs indicated that these inorganic impurities were effectively removed, which resulted in effective defibrillation of the cellulose fibers, leading to formation of micrometer long, ribbon-shaped cellulose fibers. Figure 1c exhibits the secondary electron image of extracted microfibrillated cellulose. Although there are many reports [24,25,26] on morphology of cellulose studied by a variety of techniques, secondary electron images are never reported. This manuscript reports the first-ever secondary electron image of microfibrillated cellulose. Traditionally, there are two types of secondary electron (SE) images: charge induced and ion induced. In charge-induced SE image, the lateral variations in the charge state of a sample caused by electron irradiation during and prior to image acquisition is recorded by a detector, whereas in ion-induced image, the image results from spatial inhomogeneities caused by trapped charge of ions. The SE image exhibited in Figure 1c corresponds well with the in-lens image of Figure 1b and of good quality wherein the microfibrillated fibers can be easily observed. Since the quality of SE image depends on the crystallinity and highly crystalline samples give high quality SE images, the excellent quality of the image exhibited in Figure 1c indicates that there is no drastic reduction in crystallinity of cellulose due to our NaOH/urethane system, which will be further discussed in XRD. Figure 1d is the representative TEM image of microfibrillated cellulose/NaOH/urethane aqueous solution. The TEM image exhibits an extended wormlike structure which suggests that cellulose chains exist as inclusion complexes of cellulose–NaOH–urethane aqueous solution. These extended wormlike inclusion complex typically runs into 400–450 µm in length and about 6.6 ± 0.9 µm in diameter. Moreover, the cellulose inclusion complexes are moderately dispersed in the solution, indicating that the inclusion complexes are very stable in the solution system.

The mechanism of formation extraction of cellulose by our NaOH/urethane system can be explained on the basis of formation of inclusion complex of cellulose in alkaline carbamate aqueous solutions [27]. It is widely accepted that cellulose swells in alkaline (Na or Li) solutions precooled to −10 °C, wherein alkaline Li^+^ and Na^+^ form ionic hydrates whose size and concentration depends on the solution temperature. However, in order to extract cellulose, mere swelling is not sufficient and the cellulose must dissolve. This swelling is resultant of partial dissociation of the C6-OH moieties of cellulose molecules in NaOH aqueous solution. The binding of Na^+^ ions to hydroxyl groups of cellulose ruptures the intermolecular hydrogen bonds in cellulose and leading to the dissolution of cellulose. This phenomenon is augmented by adding carbamates like urea, thiourea, and urethane, as in our case, wherein the highly polar NH_2_ groups of urethane act as hydrogen bonding donor and receptor between NaOH and C6-OH to form new hydrogen bonds, which leads to decrease in crystallinity thereby changing the molecular confirmation of cellulose from semistiff rigid structure to extended wormlike flexible chains. The chemical composition of extracted fibers as determined by the procedure described in Section 2.2 shows 87.5 wt% cellulose, of which 3 wt% is hemicellulose and 84.5 wt% is holocellulose, 6.4% moisture content, and the remaining 9.1% being lignin.

**Figure 2:**
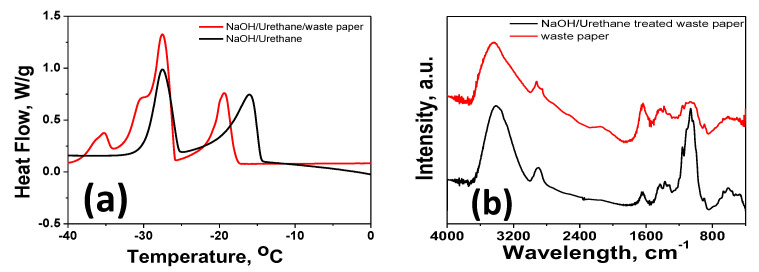
Differential scanning calorimetry (DSC) (**a**) and Fourier-transform infrared (FTIR) spectroscopy (**b**) spectra of microfibrillar cellulose extracted by NaOH/urethane/waste paper and waste paper.

In order to understand the dissolution of extraction of microfibrillated cellulose by our NaOH/urethane aqueous system, DSC study was carried out. Figure 2a presents the DSC thermograms of NaOH/urethane and 5% waste paper/NaOH/urethane. The melting peak at low temperature, around −27 °C in NaOH/urethane can be assigned to the melting of the crystalline eutectic mixture typically observed in NaOH/carbamate systems [27] and comprises one metastable sodium hydroxide pentahydrate and four water molecules (NaOH·5H_2_O; 4H_2_O) [28]. In presence of waste paper, this peak is trifurcated to three peaks at −27, −30, and −35 °C which can be assigned to melting of the crystalline eutectic mixture, the melting of free water, and urethane hydrates [29], respectively. Moreover, the high temperature peak observed at −15 °C in NaOH/urethane shows a blue shift to −20.5 °C suggesting that the urethane hydrate is possibly bound or adhered to cellulose, which results in slight variation in the structure of wormlike structure of cellulose chains. More ever, the enthalpy corresponding to the melting of NaOH hydrates in the waste paper solution increases slightly, compared with NaOH/urethane, suggesting that a certain amount of NaOH hydrates is bound to cellulose.

The FT-IR spectra of untreated and NaOH/urethane-treated waste paper is plotted in Figure 2b wherein both the samples exhibited similar spectra, which indicate that the functional groups on the paper’s surface are not drastically altered by the treatment. However, the C-H stretching peak of CH_2_ groups of cellulose backbone was slightly shifted from 2920 to 2900 cm^−1^ in NaOH/urethane-treated waste paper, which can be attributed to the partial removal of lignin and increase in the cellulose component as evident from the sharpening of asymmetric and symmetric methyl and methylene stretching groups present in the spectra of typical cellulose fiber-rich components. Besides this there was a drastic decrease in the intensity of the 1650 cm^−1^ peak, which can be assigned to stretching vibrations of aromatic C=C of lignin component in NaOH/urethane treated waste paper that further confirms our claim of removal of lignin by our alkali/urethane treatment. In typical alkali/carbamate system, though it is widely believed that NaOH interacts with hydroxyl groups of cellulose, the exact role of carbamates in cellulose dissolution is still not clear. It is believed that carbamate might play dual role of direct interaction with cellulose and/or influences the interaction between cellulose and the solvent. The chemical composition of paper is cellulose, hemicellulose, and lignin, and there are reports which suggest that the main characteristic peak of hemicellulose can be observed around 1728–1731 cm^−1^ [30]. However, in this study, we did not find any hemicellulose-related peaks in this region. Other important peak related to hemicellulose characteristic was observed at 1245–1249 cm^−1^, which was assigned to acyl-oxygen CO-OR stretching vibration in hemicelluloses prominent in the case of waste paper, whereas in NaOH/urethane-extracted microfibrillar cellulose, the intensity of this peak was reduced marginally, which indicated that a portion of hemicellulose could also be removed by our newly developed system.

**Figure 3:**
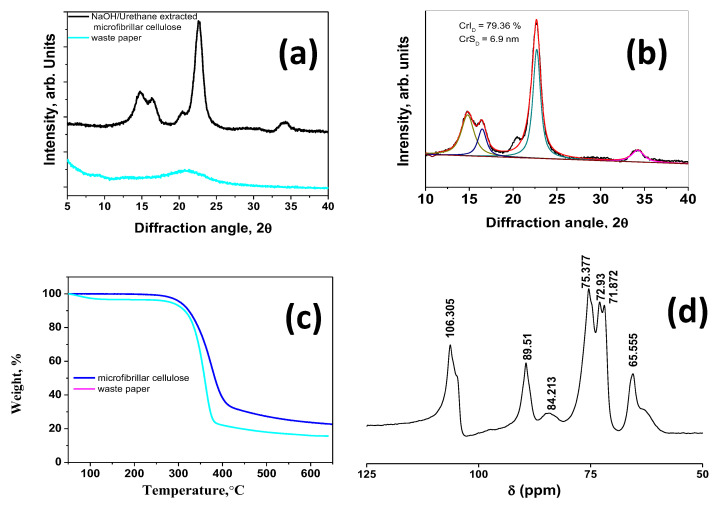
X-ray diffraction (XRD) and thermogravimetric analysis (TGA) of waste paper and microfibrillar cellulose (**a**,**c**), deconvoluted XRD spectra (**b**), and solid state 13C cross-polarization magic angle spinning nuclear magnetic resonance (CP-MAS NMR) spectrum of microfibrillar cellulose (**d**).

The X-ray diffraction spectra of waste paper and microfibrillar cellulose extracted using our NaOH/urethane aqueous system is plotted in Figure 3a. In case of waste paper, two weak peaks at 9.21 and 21.12 can be observed, whereas in case of microfibrillar cellulose, prominent diffraction peaks at 2θ = 14.8°, 16.4°, 22.5°, and 34.2° corresponding to the (1 1 0), (1 1 0), (2 0 0), and (0 4 0) crystallographic planes of cellulose I can be observed [31]. The crystallinity index (CrI) of the cellulose was estimated by using Gaussian curve fitting deconvolution method (Figure 3b) and was applied for all the four (0 0 2), (1 1 0), (110), and (0 0 4) crystalline peaks. The sharpest peaks occurring at 22.5° is typical of (2 0 0) crystalline peak of cellulose to the crystallographic plane. The degree of crystallization (CrI_D_) was calculated to be 79.36% and cellulose crystallite size as calculated by Scherrer formula was 6.9 nm, which further confirmed that our newly developed NaOH/urethane aqueous system was capable of producing highly crystalline microfibrillar cellulose. The thermal stability of microfibrillar cellulose extracted by our NaOH/urethane aqueous system as measured by TGA is illustrated in Figure 3c. The residual weight after 600 °C was 15.22 wt% in case of waste paper, whereas it was higher at 23.27 wt% in microfibrillar cellulose. From the TGA plot, occurrence of three-stage thermal degradation was observed. In the first stage, there was a 5–6% weight loss between 30 and 110 °C in waste paper due to removal of bound moisture, whereas in microfibrillar cellulose this weight loss was less than 2%. The maximum weight loss was observed in second stage of thermal degradation, which occurred in the range of 298 to 354 °C in case of waste paper and 308 to 410 °C in case of microfibrillated cellulose. The onset of degradation at lower temperatures in waste paper can be attributed to presence of hemicellulose, lignin, pectin, etc., whereas the onset of degradation at markedly higher temperature in microfibrillated cellulose can be attributed to the thermal degradation of α-cellulose, which proceeds by the incremental steps of dehydration, partial depolymerization of polysaccharides, especially the chain ends, and finally, the thermal decomposition of glycosyl units that results in elimination of hydrogen and oxygen moieties forming carbon-rich char residue [32]. The CP-MAS NMR spectrum of microfibrillar cellulose extracted by our NaOH/urethane aqueous system showed signals at δ 65.555 for the C6 atom [33], 71.872, 72.93, and 75.377 for C2, C3, and C5 carbons of cellulose, the sharp 89.51 and weak 84.213 for C4, and 106.305 for C1, which was consistent with other reports on NMR of cellulose.

**Figure 4:**
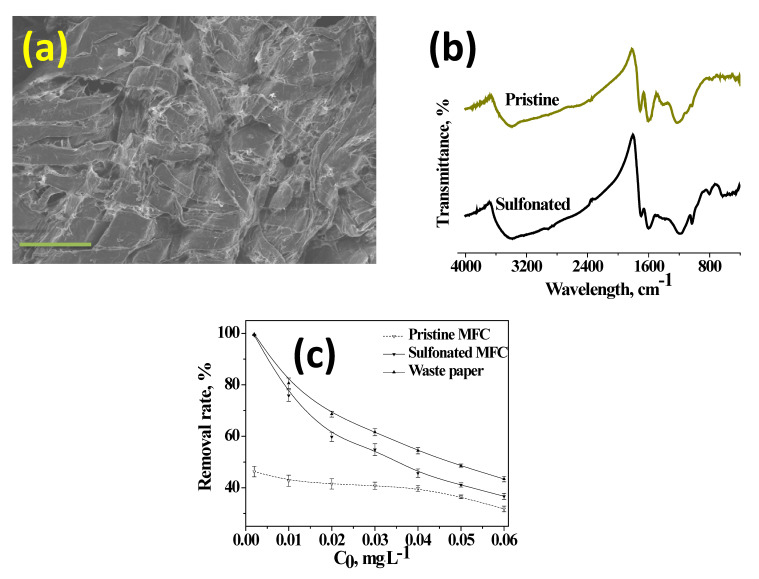
Representative SEM micrograph of sulfonated microfibrillar cellulose (**a**), FTIR spectra of pristine and sulfonated microfibrillar cellulose (**b**), and removal rate of Pb(II) at pH = 5.5 on pristine, sulfonated microfibrillar cellulose, and waste paper at various concentrations of Pb(II) (**c**).

Lead ion (Pb(II)) is the most common in drinking water due to widespread use of lead-based pipes used in domestic piping. Consumption of lead above the permissible level of 0.015 mg/L causes serious health problems like kidney malfunction, blood loss, cancer, etc. Although many techniques including membrane filtration, ion exchange, precipitation, adsorption, oxidation, reduction, etc. are in use to remove Pb(II), the most effective and economic method is adsorption. Many adsorbents both organic and inorganic can be used for removal of lead by adsorption, but naturally occurring polysaccharides and polysaccharide-derivatives like pectin [34], lignin [35], hemicelluloses [36], and cellulose [37] can be considered as effective, economical, and renewable adsorbents for wastewater treatment. Amongst these, cellulose needs special mention due to the fact that it is the largest naturally occurring polymer in the world and can be considered as inexhaustible source. However, the adsorption efficiency of heavy metals by cellulose is dependent on the surface chemistry and many surface modifications like TEMPO-based oxidation [38], phosphorylated cellulose derivatives [39], converting cellulose to carboxy methyl cellulose [40], amino functionalized cellulose [41], etc. have been reported. Microfibrillar cellulose obtained from our NaOH/urethane aqueous solvent system was sulfonated using p-toluenesulfonyl chloride in dimethyl acetamide/lithium chloride solvent system as per the protocol described by McCormick et al. [42]. The morphology after sulfonation reaction was studied by SEM. A representative SEM micrograph is presented in Figure 4a, which shows that microfibrillated cellulose retain their wormlike structure, but most of the fibers are “cross-linked” with each other forming a three-dimensional compact network. FTIR spectra were used to confirm the presence of –SO_3_H moieties on cellulose surface. The FTIR spectra of pristine and sulfonated microfibrillar cellulose plotted in Figure 4b showed a visibly discernible peak at 1057 cm^−1^ confirming the formation of –SO_3_H moieties on cellulose surface. The degree of grafting (DOG) was measured by weight gain method [43] by weighing the weight of the films before and after treatment with p-toluenesulfonyl chloride using the formula DOG, % = ((W_a_−W_o_)/W_o_) × 100 where W_a_ and W_o_ are the weights of membrane after and before treatment, respectively. From the weight gain analysis, the DOG was measured to be 8.9 wt%. Batch adsorption experiments performed to investigate the adsorption of Pb(II) on sulfonated microfibrillar cellulose films at increasing concentrations from 0.001 to 0.06 mg/L is plotted in Figure 4c. From the plots it was observed that at all investigated concentrations, sulfonated cellulose showed markedly higher adsorption capacity when compared to both pristine MFC and waste paper counterparts. This can be attributed to the presence of anionic –SO_3_H moieties, which provide active sites for capture of positively charged Pb(II) ions and retain the adsorbed lead moieties on these anchoring points. The magnitude of adsorption was higher at lower concentrations with both pristine MFC and waste paper showing almost the same adsorption efficiency, however, with increasing concentration of lead, sulfonated MFC showed better adsorption capacity. This result successfully proves that sulfonated microfibrillar cellulose can be very effective for removal of positively charged heavy metal ions from contaminated waters.

## 4. Conclusions

In summary, we report the discovery of NaOH/urethane aqueous solvent system for extraction of microfibrillated cellulose from waste paper. Morphological studies by SEM and TEM showed that tape structured, micrometer long cellulose fibers can be obtained using our newly developed technique. The excellent quality of microfibrillated cellulose fibers was confirmed using NMR and FTIR studies, whereas structural analysis by XRD showed that the cellulose fibers obtained by our technique were highly crystalline with 79.36% degree of crystallization and average crystallite size of 6.9 nm as calculated by Scherrer’s equation. The utility of pristine and sulfonated microfibrillated cellulose for removal of lead from contaminated water was also measured that showed almost 250% increase in adsorption capacity of lead by simple sulfonation.
